# Using an implementation science approach to enhance advance care planning practice: a community case study

**DOI:** 10.3389/frhs.2025.1680369

**Published:** 2025-12-04

**Authors:** Penny Lun, Chou Chuen Yu, Nongluck Pussayapibul, Sharon Straus, Siew Fong Goh, James Low, Woan Shin Tan, Raymond Ng

**Affiliations:** 1 Geriatric Education and Research Institute, Singapore, Singapore; 2 Knowledge Translation Program, St. Michael’s Hospital, Toronto, ON, Canada; 3 Department of Medicine, University of Toronto, Toronto, ON, Canada; 4 Institute of Health Policy, Management and Evaluation, University of Toronto, Toronto, ON, Canada; 5 Geriatric Medicine, Khoo Teck Puat Hospital, Singapore, Singapore; 6 Lee Kong Chian School of Medicine, Nanyang Technological University, Singapore, Singapore; 7 Palliative & Supportive Care, Woodlands Health Campus, Singapore, Singapore

**Keywords:** advance care planning, knowledge translation, knowledge to action, implementation science, stakeholder engagement, quality framework

## Abstract

The process of advance care planning (ACP) can educate and prepare patients and caregivers to make better in-the-moment end of life decisions. Two nationwide studies on ACP implementation in Singapore identified gaps and barriers across various ACP practice settings, contributing to low ACP up-take and completion rates. This case study describes the key steps on how stakeholders were engaged and supported development of a quality practice guideline for ACP implementation. A knowledge exchange platform was convened through a multi-level partnership to form a workgroup tasked to translate evidence and develop quality ACP practice guidelines. Key knowledge users such as ACP implementers were engaged throughout the Knowledge-to-Action (KTA) action-cycle phase completing various associated tasks, including an e-survey conducted to prioritize barriers identified from the nationwide studies and other relevant evaluations. Prioritized barriers and their mapped Theoretical Domains Framework (TDF) were linked with relevant intervention functions and their associated implementation strategies, then contextualized into potential local applications by the workgroup. The implementation strategies were grouped into broader categories that formed domains in the practice guideline. The last action phase was engagement with ACP teams across various organizations to conduct pilot studies using implementation strategies that would facilitate quality implementation of ACP. Our quality guideline development process was supported by the KTA model that is iterative in nature with action-phases operationalized through a multi-level partnership. This is a novel approach to formulate a national quality practice guideline with lessons learned that could be applied to others pursuing similar endeavors.

## 1 Introduction

According to the World Health Organization, the world population of those aged 60 years and above is predicted to double between 2020 and 2050 (1). A rapidly growing older population and medical advances can promote aggressive end-of-life care, which could lead to increased cost burden for individuals and societies, while not improving the quality of care or death (2). On the other hand, planning for future end-of-life (EOL) care based on one's wishes, such as through advance care planning (ACP), could better align EOL care with those wishes and improve the quality of care (3, 4). However, ACP is a complex behavioral intervention that must be tailored to the patient's life course (5). The process consists of activities ranging from an introduction to create an awareness, facilitate a discussion, document the decision, perform periodical updates, and apply the documented wishes during EOL care (6). Despite resources invested into ACP programs (7) and overall positive views of ACP from healthcare professionals and patients (6), adoption of ACP remains low (8–11).

Among the factors that contribute to the low adoption of ACP, variations in its definitions, scopes, and heterogeneity of outcome measures targeting patients, caregivers, providers, or processes have added challenges to its successful implementation and uptake (5, 6, 12). Key implementation challenges include supporting the value of ACP with its non-legal binding framework within health system and institutional-level policies, aligning with the cultural and racial values of patients and caregivers, as well as activating ground level service providers who implement the ACP process (6). An overview of systematic reviews identifies implementation barriers and facilitators at the patient, caregiver, health care provider, institutional, and operational levels (6). For example, patient and caregivers tend to lack readiness to talk about advance care decisions and prefer initiation of such discussions by the attending healthcare professionals (6, 13). On the other hand, healthcare professionals (HCPs) are generally hampered by a lack of knowledge and skills to initiate or conduct ACP, or having limited time to engage in such conversations (6, 13). Improving their skills and attitudes would enhance the quality of how ACP is implemented as a process, which could reduce the barriers experienced by patients and their families (6, 13). In addition, country-level contexts and cultures additionally play into how ACP is interpreted and implemented, highlighting the need to take a localized approach (6).

With the growing understanding that ACP should be part of the continuum of care through international and regional Delphi panels (4, 14–16), a care planning umbrella framework that focuses on preparing for communication and making medical decisions was proposed (17). In this framework, care preparation would extend across the healthy, the chronic disease, serious illness, and EOL states (17). At each stage of health and illness, in-the-moment and advance decisions should be iteratively explored as they are mutable over one's life course (17). As the process of ACP is complicated, understanding the barriers and facilitators during stages of the implementation process is crucial. Implementation science methods could point to tailored strategies that work in specific contexts and improve the overall implementation quality. This would help to disentangle the complexities without losing sight of how each outcome domain is interlinked with and impact each another (12, 17). Hence, a “whole-system strategic approach” should be considered (6).

## 2 Context

### 2.1 Overview of ACP program in Singapore

The national ACP program named “Living Matters” in Singapore was started in 2011 and modelled after the Respecting Choices Program from the Gundersen Lutheran Health System in Wisconsin, USA (18). The need for ACP had evolved from the shortcomings of legal approaches such as the Advance Medical Directives (AMD) in general and in Singapore for its poor uptake (19, 20). The Mental Capacity Act that was passed in 2008 emphasizes the need to take one's known preferences and wishes into healthcare decision making, setting the stage for ACP (21). The aim of ACP is to empower patients in shared decision-making in care goals near EOL and to improve the quality of palliative care (22, 23). It is not legally binding as it is meant to be an ongoing conversation that account for changing preferences over time (24). Spearheaded by the National ACP Steering Committee that was appointed by the Ministry of Health (MOH) and helmed by the Agency for Integrated Care (AIC), an implementation arm of the MOH, the “Living Matters” program was rolled out in two phases. The first phase was in inpatient care within public hospitals, followed by a second phase at outpatient care within the public hospitals, in the primary care polyclinics, and in nursing homes (23, 25).

The program is operationalized through the disbursement of ACP program fundings, the development and maintenance of a national information technology system to capture completed ACP documents (21), administration of a two-part ACP facilitator training curriculum and certification (26), and standardization of ACP forms and documents. However, each organization receiving the program funding is able to implement ACP in a contextually suitable manner. In Singapore, healthcare is operated through three regional integrated clusters with each responsible for the health and well-being of the population living within certain geographic boundaries (27, 28). Each cluster consists of services across the care spectrum from primary care to long-term care facilities (28). This inevitably has led to variable practices within and across settings (22).

Local studies have pointed to various challenges and barriers from system-related problems, such as the lack of leadership or low organizational priority that carried through to low acceptance among staff. Given that ACP is non-legally binding, physicians may override patients' documented wishes when their decisions are deemed in best medical interest, organizational leadership support could help to ensure a culture that prioritizes patients' wishes and preferences during their end-of-life care (22). On the other hand, challenges were also impeded by patients or families rejecting ACP discussions and difficulties in retrieving ACP documents when needed (22, 23, 29). In addition, barriers among healthcare professionals remain, such as misconceptions on ACP functions and competing priorities of clinical duties contributing to why ACP introductions were not initiated, let alone completed (21).

### 2.2 Motivation for change

Two national evaluations in 2018 and 2021, respectively, found implementation inconsistencies and gaps across organizations and settings (30, 31), which in part contributed to the low take-up and completion rate. The list of barriers and challenges to implementation spanned across macro-, meso-, and micro-level issues. Setting quality standards in ACP implementation and practice was identified as needing immediate attention.

Hence, this case study describes how the evaluation reports motivated ACP implementers to drive an improvement with the goal to “strive for and adopt certain best practices, thereby strengthening ACP implementation across all organizations” (31). The focus will be on key steps of how an implementation science approach is operationalized to support co-creation of a national quality practice framework.

## 3 Implementation science approach: engagement and translation

### 3.1 Aims and formation of the multi-level stakeholder partnership

To ensure that the evidence found in the evaluation reports translate into quality practice nationally, a knowledge exchange platform with multi-level partnership was convened. A knowledge translation or exchange platform aims to narrow the gap between evidence and practice by bringing the potential knowledge-users or implementers (e.g., policy-makers, practitioners, researchers) together through dialogue to produce context-relevant evidence-informed practice, policies, or programs (32, 33). Our knowledge exchange platform was guided by the Knowledge-to-Action (KTA) cycle, a conceptual model based on planned action theories that maps translation of evidence into real-world practice, emphasizing inclusion of knowledge-users from the start (34, 35). From the beginning, the key aims of our knowledge exchange platform were to develop a national quality practice framework and to increase its future adoption by engaging and activating practitioners through the iterative principles of implementation science, imparting KTA cycle knowledge along the process (36, 37).

**Figure 1:**
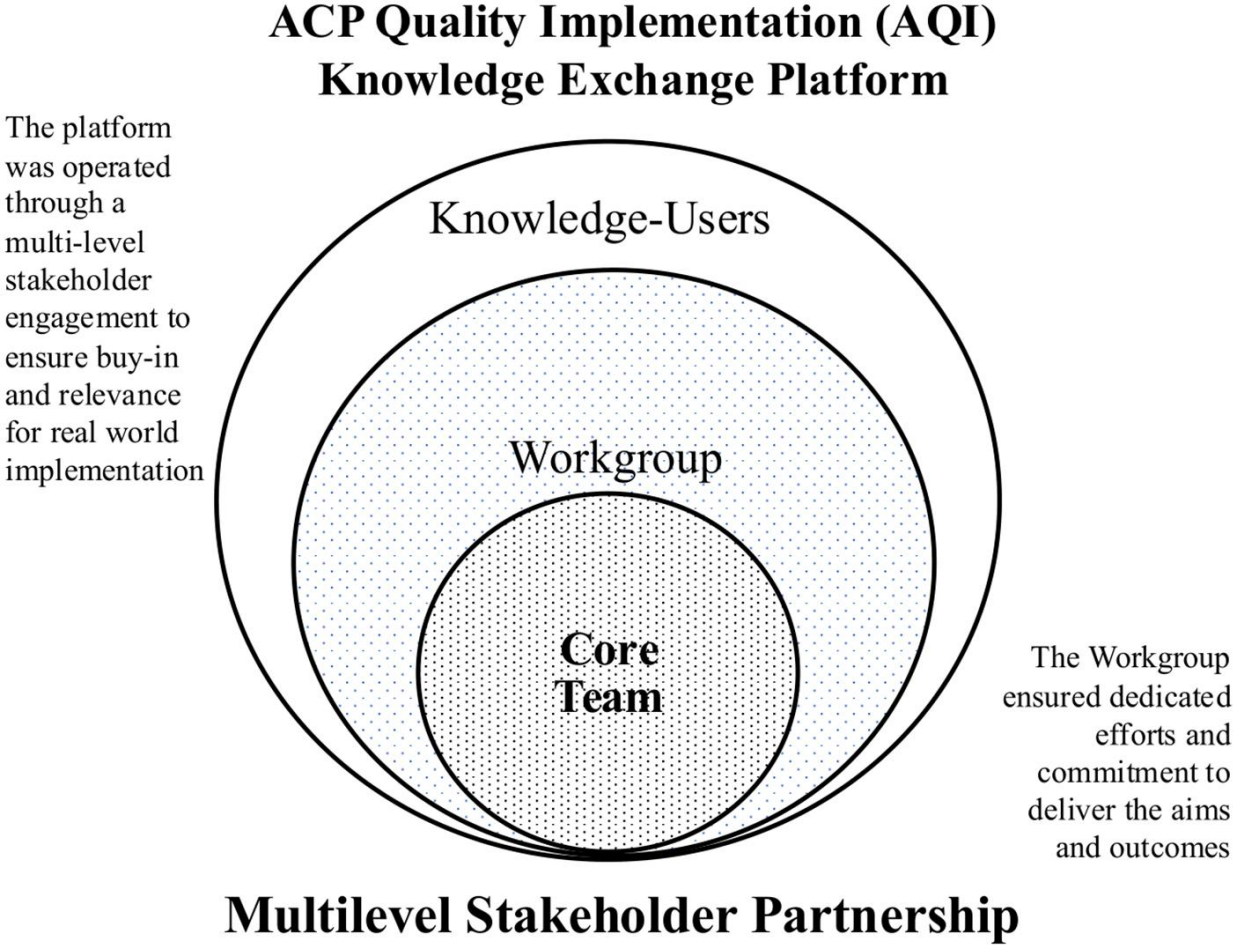
Multilevel stakeholder partnership.

A core team comprising 14 representatives from the National ACP Steering Committee (Chairperson), domain experts from AIC, clinicians, researchers, as well as administrators with implementation science knowledge, was formed initially to lead the tasks. Remaining members of the workgroup were sampled from the three healthcare clusters based on their cadre in ACP leadership and implementation roles, as well as professional background (*i.e*., physicians, nurses), resulting in an additional 14 members joining the Quality Implementation (AQI) Workgroup (21). Figure 1 shows the various stakeholder groups involved as part of the knowledge exchange platform.

A kick-off meeting of the workgroup on 11 Jan 2023 introduced the group and the aims, as well as set the stage for how to achieve them within two years. The core team led the engagement to deliver the aims and outcomes, including identifying and applying methodology in the process, including consultations with a knowledge translation (KT) pioneer. Prior to each workgroup meeting, tasks and agendas were discussed and deliberated by the core team before being presented to the workgroup for collective decision making.

### 3.2 Engagement and knowledge translation through the knowledge-to-action model

**Figure 2:**
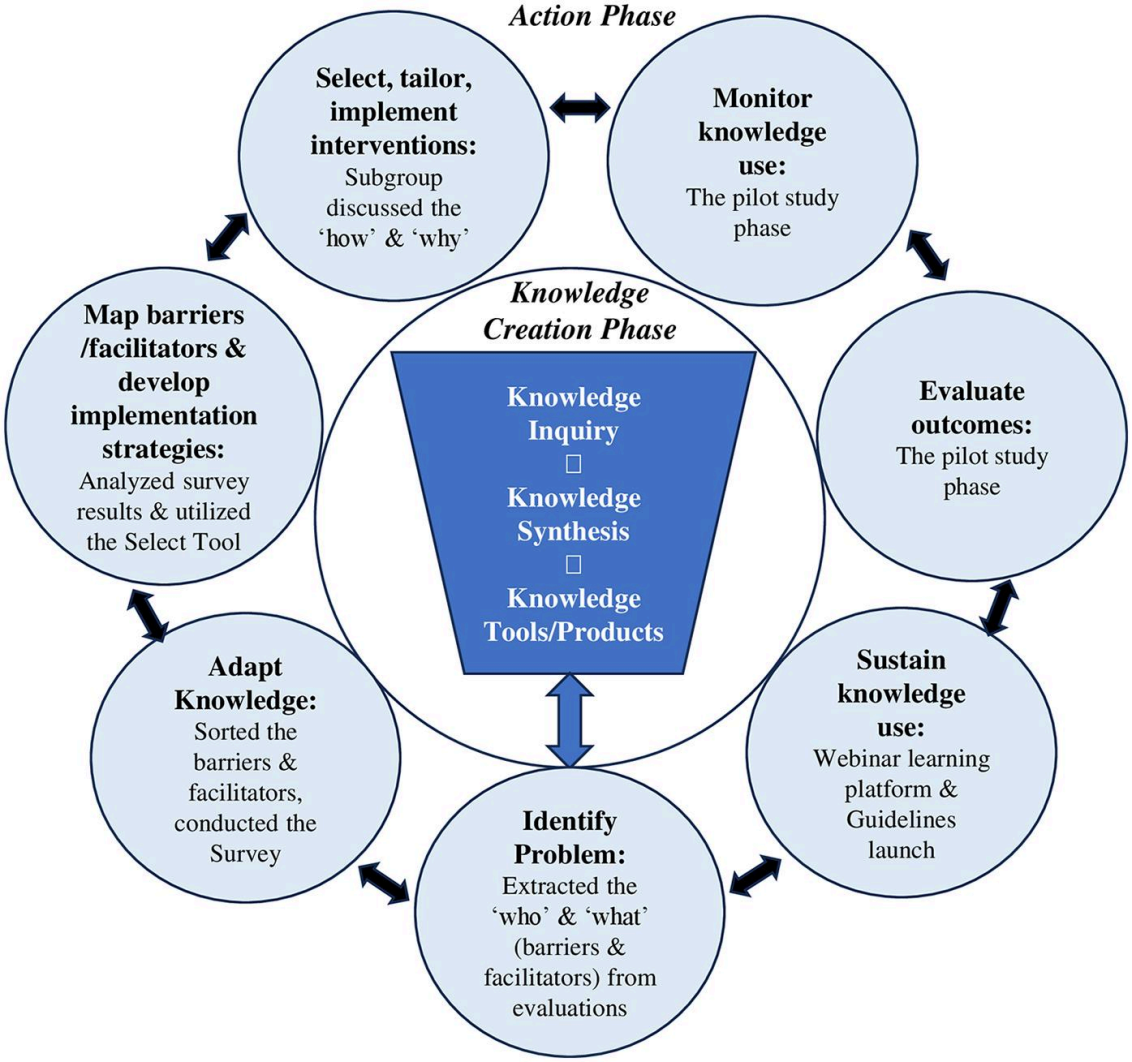
Key framework development process in the Knowledge-to-Action Framework.

The Knowledge-to-Action (KTA) model provides a structured, planned-action approach and was well-suited to our purpose to effect changes in the complex ACP ecosystem through the platform and workgroup (34). Various knowledge-users were engaged at different timepoints of the project to serve diverse purposes along the KTA processes. The KTA cycle consists of an inner funnel that is the knowledge creation phase, and the outer circle that depicts the seven action phases where evidence is translated into practice (34, 35). The action phase is based on planned-action theories to cause deliberate changes in groups (34, 35). Our engagement process, actions, and outputs are detailed through the action phase that contains knowledge application activities (34). Figure 2 shows the adapted KTA cycle depicting the key framework development process. Supplementary Figure S1 shows the KTA cycle that guided the process (34, 35).

#### 3.2.1 Identify problem

The first step of the action phase involves identifying, reviewing, then selecting knowledge for implementation (34, 35). Local evidence derived from the two existing national evaluation reports (30, 31), an ACP facilitator training evaluation report (26), and focus group findings from community ACP facilitators (38) were examined to guide formulation of the quality practice framework. These documents identified the known scope on the “Who” (ACP facilitators, healthcare and community-care professionals, patients/caregivers) and the “What” (barriers and facilitators) to ACP implementation in Singapore. In our context, barriers are defined as factors that hindered the ACP implementation process and prevented its completion, while facilitators are factors that promoted the ACP implementation process that led to successful completion of ACP sessions and documentation.

Barriers and facilitators from the documents were extracted, synthesized, and mapped to 14 domains in the Theoretical Domains Framework (TDF) by two domain experts independently and cross- checked by a researcher in the AQI workgroup. TDF is an implementation framework synthesizing 33 theories of behavior and behaviour change into 14 domains of influences on the behaviors of health professionals when implementing evidence-based recommendations (39, 40). For example, under the domain of “knowledge”, lacking knowledge on ACP might prevent a healthcare professional from initiating an ACP conversation with a patient, which would result in that patient missing a chance to consider ACP. The domains are linked to intervention functions with implementation strategies that could be utilized to promote uptake of practice (39–41).

#### 3.2.2 Adapt knowledge to local context

Adapting knowledge to the local context involves going through a process to determine if certain knowledge is useful or appropriate in a context (34, 35). We operationalized this step via several steps. We first sorted through the extracted list of wide-scope barriers and facilitators and categorized them into system-, client-, and ACP process-related (pre-, intra-, post-ACP) levels for easier curation. In our context, pre-ACP process refers to activities that need to take place before the actual conduct of an ACP session. For example, the ACP-Facilitator training and certification that prepares one to conduct a session and the referral process for ACP. Intra-ACP process refers to conducting of the ACP conversation/discussion by a certified ACP-facilitator, completing, and uploading of the ACP document to the national ACP portal. Post-ACP refers to situations (*i.e*., EOL care) when ACP documents are retrieved and consulted for care decisions. Next, we aimed to engage relevant knowledge-users to better understand how common the scoped barriers were encountered at respective ACP practice sites. A national survey targeting all ACP-trained facilitators to help prioritize the barriers was a way to engage the wider knowledge-users in the practice framework development process.

##### 3.2.2.1 The survey

Sampling frame for the survey consisted of certified ACP-facilitators from the national ACP registry and ACP representatives reached through targeted cold-calls to nursing homes and community hospitals, amounting to around 5,000 email addresses for this ACP implementer group. Based on suggestions made by some of the workgroup members, HCPs who were not trained or certified as ACP-facilitators but contributed to the implementation process by making referrals or executing ACP-informed care plans were included. Purposive sampling was adopted to identify these HCPs through the ACP organizational clinical leads, and it was up to each lead to determine how many staff were reached.

Survey questions were formulated based on the barriers identified in the evaluation documents (26, 30, 31, 38). Similar barriers were combined through discussions within the core team. The fully drafted survey was subsequently circulated with the rest of the workgroup for comments, resulting in 58 statements categorised by levels (system-, client-, process-related). Survey participants were asked to rate their level of agreement on whether they had experienced the barriers within their organizations using a 5-point Likert scale with anchors ranging from strongly disagree-1 to strongly agree-5. A mean score for each barrier statement was derived and a higher score indicated stronger endorsement of the barrier. Email invitations with a Qualtrics survey link were sent to the groups in mid-March 2023 and they were given a month to respond, with two staggered reminders sent over that period. Personal identifiers were not collected in the survey. Supplementary Table S1 shows the breakdown of survey sections for the participant groups.

A total of 584 (∼11%) completed responses were received with overall representation by professional backgrounds (doctors, nurses, social workers or others who were certified ACP-facilitators or not ACP-trained) and by practice settings (primary care, acute hospitals, community hospitals, nursing homes, home care and community). Most of the participants were female (78.1%), with a mean age of 42.6, and on average 11.8 years of professional experience. Characteristics of the survey participants are shown in Supplementary Table S2.

The top 25% of barriers prioritized by the ACP implementers spanned across system, client, and intra-ACP process levels. System and client level barriers made up 80% of the list, while the remaining 20% were intra-ACP process-related barriers, signaling issues with overall resource allocation in ACP implementation, as well as challenges with engaging a wider target ACP audience. Table 1 shows the top barriers experienced by the implementers. Supplementary Table S3 shows results of the remaining barriers by levels.

**Table 1:** Top 25% of barriers identified by ACP implementers (*n* = 486).

Level	Barrier	Mean	SD
System	Output-driven key performance indicators do not adequately measure efforts	3.86	.90
Client	Low awareness of ACP among general public	3.86	.80
Client	Lack of enduring outreach strategies to general public	3.77	.79
Client	Lack of knowledge on disease trajectories, treatment, and prognosis	3.73	.80
Client	Lack of targeted outreach to minority communities	3.66	.80
System	Lack of ACP facilitators for minority communities	3.64	.84
System	Low priority as facilitators juggle multiple commitments	3.63	1.09
System	Lack of protected manpower, time and resources	3.59	1.07
Process: Intra-ACP	Lack of medical knowledge a barrier for non-clinically trained facilitators	3.54	.89
System	Lack of clear career progression for full-time facilitators/trainers	3.53	.90
Process: Intra-ACP	Lack of fluency in local languages hinders ACP completion	3.49	.92
Client	Lack of ACP acceptance among general public	3.48	.91
Client	Avoid discussing about end-of-life care priorities	3.45	.89
System	Lack of incentives for staff and department to adopt ACP	3.42	1.00
Process: Intra-ACP	Find ACP documentation to be tedious	3.38	1.01

#### 3.2.3 Map barriers/facilitators and develop implementation strategies

This step was adapted from the assessing barriers/facilitators KTA action phase for the purpose of our process. Researchers on the core team analyzed the survey results and mapped the barriers to relevant implementation strategies. We utilized the Select Tool that is designed with steps to support implementation efforts in operationalizing theory-driven strategies (42). The barriers were linked to intervention functions and the accompanying implementation strategies through the mapped TDF domains (42). As each TDF domain contains more than one intervention function, a systematic way to sort through them is needed.

Due to the numerous barriers, we adapted a step in the Select Tool (42) to sort and prioritize the intervention functions by group (system-, client-, and process-related barriers), with higher scores indicating higher priorities for implementation. For example, for the intra-ACP process level barriers, intervention functions such as *Education* and *Enablement* had the highest occurrences, which signaled the need to adopt related implementation strategies. Table 2 shows the prioritized intervention functions in each level of the barriers with our adaptation. Next, relevant implementation strategies in the Select Tool and the Expert Recommendations for Implementing Change (ERIC) project (43), and the extracted facilitators in the earlier process, were compiled and shared with the workgroup via email. The facilitators were meant to provide a reference on what worked in some local contexts. To provide a full picture for the workgroup, all barriers differentiated by prioritized categories (Top 25%, mean ≥ 3, and mean < 3) were presented with respective intervention functions and implementation strategies.

**Table 2:** Prioritized intervention functions at each level of barriers [adapted from the select tool (42) step 2].

Intervention functions^a^	System-level	Client-level	Process-level		
			Pre-ACP^b^	Intra-ACP	Post-ACP
Coercion	4	3	4	2	2
Education	4	3	7	4	4
Enablement	5	5	7	4	4
Environmental Restructuring	4	4	3	2	2
Incentivization	4	3	4	2	2
Modelling	3	2	3	0	1
Persuasion	4	3	4	2	2
Restriction	1	2	0	2	1
Training	1	1	4	2	2

a Higher scores indicate higher priority for consideration.

b Including HCPs (non-ACP).

#### 3.2.4 Select, tailor, implement interventions

This step involves making decisions on implementation strategies that would effect changes and to tailor them to the practice context (34, 35). Our goal was to engage the workgroup members who have direct ACP implementation experiences to derive a list of potential strategies that are feasible in the local context, taking the compiled intervention functions, implementation strategies, and facilitators into consideration. The workgroup was divided into four subgroups with each focused on a respective level of the barriers (*i.e*., system, client-related levels) during their online meetings. Each subgroup consisted of an appointed leader and was supported by members of the core team for guidance and facilitation during the process. They were asked to include the prioritized barriers from the survey and to consider the non-prioritized ones for potential inclusion. During the online meetings, subgroup members were engaged to discuss whether a strategy would be feasible in their practice settings in terms of resources and cultural norms or nuances. They were then asked how to tailor the strategies. The last task was to group similar strategies together and to derive broader categories which helped to structure the domains in the quality practice framework.

The subgroup discussions enriched our understanding of the survey results by contextualizing the “why” and suggesting the “how” that might work in the local context. Results from the four subgroups were subsequently consolidated by the core team and shared with the workgroup. Table 3 shows the adapted steps from the Select Tool (42) using an example barrier.

**Table 3:** Adapted steps from the select tool (42) using an example barrier.

Barrier level	Process: pre-ACP
Barrier statement	Most of my ACP clients and their families are not emotionally ready to talk about death and dying
TDF Domain	Beliefs about consequences
Intervention functions	Coercion, Education, Incentivization, Persuasion
Prioritized intervention functions	Education, Enablement
Relevant implementation strategies (Select tool & ERIC)	Hold meetings or outreach involving program targets to improve knowledge about ACP (education). Identify and prepare individuals in the community (e.g., those who have positive experience with ACP) to dedicate themselves to supporting, marketing and overcoming indifference or resistance related to ACP (enablement).
Facilitators	Personal experiences of loss/suffering may trigger ACP completion. Personal beliefs held implicit assumptions about the nature of mortality; the underlying concept being that death is a “natural” and unavoidable part of life. Clients’ personality traits of being “open-minded”, independent and “positive” in their social interaction were positive drivers towards ACP.
Target audience	Provider
subgroup's input	ACP client screening - Incorporation of screening questions for referring HCP before referral Preparing ACP clients for ACP conversations - Pre-ACP appointment call to ACP clients: Checking client's understanding of purpose of appointment and what is ACP before actual appointment Having the ACP conversation - Contextualisation of ACP - Potential clearer referral/escalation processes - Slower pace of conversations (e.g., spaced conversations) - “Light - touch” ACPs Recognising ongoing ACP conversations - Recognise “light touch”/incomplete/partial ACP conversations where focus is on values and goals of family and ACP client ACP follow-up - Referral process should also have a follow-up/feedback loop if ACP client is not keen for ACP now (e.g., EMR notifications to physicians to follow-up in subsequent appointment, sending of brochures to clients, reminder to HCPs to follow up with clients a few months after initial ACP initiation) ACP facilitator training - Death/dying readiness survey as part of ACP facilitator training - Training facilitators to conduct “light touch” conversations (e.g., scenario for ACP facilitators to practice on and potential escalation strategies)
Broad categories (domains)	ACP client screening Preparing ACP clients Having the ACP conversations Recognising ongoing ACP conversations ACP follow-up ACP facilitator training

#### 3.2.5 Monitor knowledge use & evaluate outcomes

Monitoring knowledge use during the implementation stage will provide information on its adoption as well as observing if desired changes happened through evaluating outcomes (34, 35). Our process in this step started with the introduction of the drafted framework to the workgroup during a meeting in October 2023. We also presented a pilot study phase as the next step in the process, with the goal of rallying workgroup members to form or engage quality improvement (QI) teams in their respective practice sites and to start utilizing the implementation strategies. As most of prioritized barriers would require longer term planning and implementation, the core team suggested a curated list of barriers and associated implementation strategies that could potentially be addressed within a six-month proposed timeline. Nonetheless, it was entirely up to the QI sites to pick the most relevant barrier(s) in their contexts.

The goal of the pilot study phase is to soft launch the framework and to introduce the KTA model to potential QI teams, approaching the change mechanism through managing barriers with evidence-based implementation strategies. This was also an opportunity to engage healthcare and community care teams in the practice change process. Over the pilot study period, the core team provided consultation support on implementation strategies, but the QI pilot study teams followed their respective site QI procedures. In early November 2023, a formal invitation email was sent by the ACP National office to the workgroup members to help disseminate the AQI work within their organizations. Subsequently, eight QI teams from five acute hospitals registered interest to participate. The barrier(s) picked were related to ACP processes (pre-, intra-, post-ACP), with several teams focusing on addressing various barriers that thwarted initiation of ACP conversations.

A kickoff meeting in-person or via ZOOM was held for each QI team between December 2023 and February 2024. Reading materials on behavior change interventions and the KTA model were shared with the teams prior to the meetings. The one-hour kickoff meetings were delivered by members of the core team with the following agenda: briefing on the AQI workgroup aims and draft framework, introduction to knowledge translation principles, a discussion session on their identified problem and context of practice, and introduction to an implementation action plan (IAP). The IAP form used in the study was based on the training manual by the Knowledge Translation Program at St. Michael's Hospital (44). Following the meetings, the QI teams received support from the core team on formulation of their IAPs using evidence-based strategies tailored to their identified barrier(s), along with respective measurable outcomes. Most teams aimed to increase the initiation of ACP conversations among their clients or the number of completed ACP sessions. An implementation science pioneer was also consulted on the feedback to the teams. The core team remained available for consultation with the QI teams during the duration of the teams' efforts in implementing their QI projects through 2024.

#### 3.2.6 Sustain Knowledge Use

This step is setting a plan on how to sustain use of the knowledge long term (34, 35). A quality improvement learning platform was organized as a webinar on 9 October 2024 where five of the QI teams presented their projects to share their experiences, evaluation on improvements achieved, and key lessons learned in the process. By engaging the knowledge-users to share their learnings, we hoped that their testimonies would inspire others to make necessary changes to how ACP is implemented at their practice sites. The webinar was attended by 610 attendees from across the three healthcare clusters, community care, educational institutions, and government sectors. Ninety-five percent of the 215 who completed a post-webinar survey agreed that the sharing was useful and could be translated into practice. The framework was finalized in December 2024 and renamed to “Guidelines for quality implementation of ACP”. It was reviewed by the work group and then endorsed by the National ACP Steering Committee in March 2025, before being circulated to the Ministry of Health. Subsequently, it was officially launched by AIC in May 2025 through webinars and a community seminar with the e-guideline available through download.

## 4 Discussion

This case study describes a novel approach of using an implementation science method that emphasizes engaging knowledge-users in a knowledge translation process to improve the quality of ACP implementation in Singapore. The two-year co-creating effort through the AQI workgroup not only saw the development of a national practice guideline, but also the formation of relationships and partnerships among the ACP implementer community through various engagement activities, culminating in a learning platform that showcased pilot implementation by QI teams from various organizations. The KTA model is dynamic and iterative, providing a systematic guidance along our process from barriers and facilitators to context-relevant implementation strategies. Here we share observations and lessons learned and our reflections on the methodological constraints experienced in the process.

### 4.1 Reflections and lessons learned

#### 4.1.1 Multi-level stakeholder engagement process

Engagement of the workgroup through the KTA process required significant commitment over a two-year timeframe. Identifying strategic partners and stakeholders who believed in contributing to the national effort to drive improvements in the implementation of ACP during the initial knowledge exchange platform setup was a crucial first step. Assembling a core team with diverse policy, practical and research experiences and skills in the national ACP program was an advantage. It enabled an efficient coordination and delegation of the tasks among the members. Due to the diversity of team members, our views on the ways forward were not always aligned, but agreement on the end goals helped the core team to stay focused on the why of the endeavor. Engagement skills in communicating rationales and ensuring transparency and objectivity of the KTA process were vital to navigate individual or organizational perspectives, different mental models, and expectations.

In addition, the iterative nature of the translation work required flexibility and the ability to shift timelines to accommodate the knowledge-users. The core team found ways to overcome a shortened timeline to complete remaining tasks in order to keep to the two-year project timeline. For instance, the core team curated a list of barriers and implementation strategies that potentially required a shorter runway to complete, due to a later start to the pilot study phase.

#### 4.1.2 Application of the KTA model

Overall, the KTA model provided a systematic approach that supported our multi-level stakeholder engagement practice framework development process. It supported knowledge translation and exchange between policy makers, researchers, ACP leaders and administrators, and ACP implementers. Although most of us were learning or applying the KTA model for the first time, the core team benefitted from having a KT pioneer on board to guide the process. Core team members actively imparted KT concepts throughout the AQI project during workgroup meetings and during wider ACP community engagement activities over the two years. This was necessary as the implementation science is nascent in the ACP work in Singapore.

During the quality guideline development phase, our application of the KTA model seemed somewhat linear, but the model is meant to be iterative and flexible in its application (34, 35). For example, a nursing education and practice model was developed from the knowledge created through a doctoral work and was continually refined and tailored via engaging stakeholders in education, training, practice, and feedback in the process (45). These activities iterated between the steps of adapting knowledge to local context and selecting, tailoring, and implementing interventions in the KTA action-cycle (45). Likewise in our context, future feedbacks or evaluations from organizations that adopt the guidelines will provide insights on the practical use. This could be achieved through ACP implementers utilizing respective implementation strategies and evaluating the persistent or “new” barriers encountered during rounds of implementation with refinement each time. For sustainment going forward, it would be helpful to have regular learning platforms for these knowledge-users to share their experiences, in order to shorten the learning curve for others who are implementing similar strategies. Additionally, these learning and sharing platforms would also contribute to refinement of the national ACP practice guidelines in the long term.

While the KTA model broadly and systematically guided the translation steps, it was also necessary to adopt theoretical frameworks and tools, such as the TDF and the Select Tool in our case, to translate the barriers into appropriate implementation strategies. As we had adapted the Select Tool to better fit our purpose by grouping the barriers into broader levels (system, client, process-related), the prioritized intervention functions identified in the Select Tool did not align well with some barriers. For this reason, the “default” intervention functions for some individual barriers remained more relevant over the group-level “prioritized” intervention functions. Ultimately, the option was for ACP implementers with ground implementation experiences to consider what would work in their contexts. Hence, it is important to recognize and accept limitations that might arise from adaptation of the Select Tool.

### 4.2 Conceptual or methodological constraints

Involving stakeholders, such as knowledge-users, to co-create practice guidelines has the advantages of their buy-in from the beginning, but there are limitations of note that should be reflected for future reference. Since one of the aims of the knowledge exchange platform was to impart implementation science knowledge, the core team needed to strategize on how to introduce the concepts, steps, and tasks of the KTA cycle during the series of workgroup meetings.

The core group adopted several strategies, some of which might in turn have conceptual or methodological impact on the framework. The KTA model, with its action phases and tasks, was progressively introduced over the workgroup meetings. Many hours of curating the evidence and information to the essential and minimum amount took place prior to each workgroup meeting, including sub-core team and core-team discussion meetings. The goal was finding a balance between providing enough information for informed decision-making but not overwhelming the knowledge-users and risking disengagement. This strategy was adopted throughout the engagement process with all the stakeholders for pragmatic reasons. Formulation of the survey questions underwent the same process. As a result, we might have missed information that the stakeholders would have otherwise considered important.

Due to the fact that our workgroup members and other stakeholders are busy healthcare or community care professionals with competing priorities, it was not realistic to expect the same level of engagement throughout the engagement period or high engagement for single time-point engagements. For example, there was a greater representation in the survey from “key knowledge-users”, such as full-time facilitators, trainers and mentors, vs. the other ACP-trained HCPs who were only conducting ACP sessions on an ad-hoc basis. The latter group might have experienced different barriers that prevented them from initiating or conducting ACP in general.

## 5 Conclusion

This is a case study that describes how an implementation science approach was used to support development of a practice guideline to improve ACP implementation. By utilizing the KTA model, it systematically guided the multi-level stakeholder engagement process to derive a set of practice guidelines for quality ACP implementation in Singapore. Involving relevant stakeholders ensured buy-in on the guidelines, but a real challenge remains on adoption and sustainability of the practice guideline in an environment where other higher-prioritized problems are competing for improvement opportunities. Adoption of the practice guidelines at the national policy level and by various organizations can help to raise awareness of the importance of the ACP process, as well as improve the quality of its implementation, including addressing system and client-related barriers. The ACP process is a complex and multi-dimensional endeavour that must be adapted to each culture and context. Application of implementation science principles can help foster the full potential of ACP implementation in real world settings.

## Data Availability

The original contributions presented in the study are included in the article/Supplementary Material, further inquiries can be directed to the corresponding author.

## Supplementary material

Supplementary Figure S1 Knowledge-to-Action Framework. Reproduced with permission from Graham et al. (34).
