# Supplementary material for: Using an implementation science approach to enhance advance care planning practice: a community case study
Source: Front Health Serv. 2025 Dec 4;5:1680369. doi: 10.3389/frhs.2025.1680369 (PMC12711861; doi:10.3389/frhs.2025.1680369)
Supplement: Supplementary Figure S1 — Knowledge-to-Action Framework. Reproduced with permission from Graham et al. (34). [file Datasheet1.docx]

**Supplementary Material**


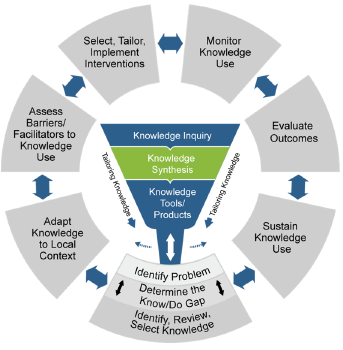


Figure S1. Knowledge-to-Action Framework

Table S1: Breakdown of survey sections for participant groups

| **Survey sections & sub-sections**  **(No. of barrier statements)** | **ACP* Implementers** | **HCP****  **(Non-ACP)** | |
| --- | --- | --- | --- |
| Demographics: Age, practice setting, sex, professional role, years in profession, primary role in ACP  Total years of experience in ACP  Estimated ACP completed in past 1 year | √  √  √ | | √  -  - |
| **System-related Barriers** | | | |
| Institutional leadership and resourcing (8) | √ | | - |
| Cross-institutional workflow integration (1) | √ | | - |
| Governance and service (2) | √ | | - |
| Care preferences (1) | √ | | - |
| Key performance measures (1) | √ | | - |
| **Client-related Barriers** |  | |  |
| Public life and death education (1) | √ | | - |
| General Outreach (3) | √ | | - |
| Targeted outreach (1) | √ | | - |
| Health Literacy (2) | √ | | - |
| Trust to the healthcare system (1) | √ | | - |
| Filial Piety (1) | √ | | - |
| Caregiver support (1) | √ | | - |
| **Process barriers-Pre ACP** |  | |  |
| Referral process (4) | √ | | √ |
| Preparatory training (3) | √ | | - |
| **Process barriers- Intra ACP** |  | |  |
| Engagement skills (5) | √ | | - |
| Dissonance (1) | √ | | - |
| ACP conversation and documentation (4) | √ | | - |
| ACP Documentation and IT infrastructure (4) | √ | | - |
| Family dynamics (1) | √ | | - |
| **Process barriers- Post ACP** |  | |  |
| Work-flow (1) | √ | | √ |
| Retrieval (4) | √ | | √ |
| Value of ACP (3) | √ | | √ |
| Prioritizing of Themes (22) | √ | | - |
| Feasibility of a referral (2) | - | | √ |
| At odds with values/roles (2) | - | | √ |
| Expectations of clients/ families (4) | - | | √ |

**ACP Implementers: All full-time and part-time certified ACP facilitators who are doctors, nurses, social workers or others. **HCP(Non- ACP): Healthcare professionals who do not have specific ACP implementer roles, but would refer suitable patients for ACP or execute ACP-informed care plans*

Table S2: Characteristics of the survey participants

| Overall (n= 584) | |  | |
| --- | --- | --- | --- |
| Gender | Female | | 456 (78.1%) |
|  | Male | | 127 (21.9%) |
| Age | Mean | | 42.6 years old |
| Years in current professional role | Mean | | 11.8 years |
| Professional role | Doctors | | 120 (20.5%) |
|  | Nurses | | 261 (44.7%) |
|  | Hospital Social Workers | | 77 (13.2%) |
|  | Full-time facilitators | | 27 (4.6%) |
|  | Others (*e.g*., community Social Workers, Care mangers/coordinators) | | 99 (17.0%) |
| Settings* | Primary care | | 45 (7.7%) |
|  | Acute hospital | | 185 (31.7) |
|  | Community hospital | | 29 (5.0%) |
|  | Nursing home | | 219 (37.5%) |
|  | Home care | | 37 (6.3%) |
|  | Community | | 68 (11.6%) |
|  | Others (e.g., dialysis centers, hospice, day care, group homes) | | 25 (4.3%) |
| ACP implementers: facilitators, trainers, mentors  (n = 486) | | | |
| Years in ACP implementation | Mean | | 4.2 years |
| Number of ACP completed in the past 1 year | Mean  Median | | 11  3 |

**Some practise at more than one setting*

Table S3: Mean scores of remaining barriers by levels

| Barrier | Groups* | n | Mean | SD |
| --- | --- | --- | --- | --- |
| System |  |  |  |  |
| Lack of common referral process and work processes | ACP Implementers | 486 | 3.28 | .96 |
| Lack of strong leadership at the national policy level | ACP Implementers | 486 | 3.21 | 1.01 |
| Fixation on cure may override client’s preferences | ACP Implementers | 486 | 3.18 | .95 |
| Lack of community-based care services to support honouring wishes | ACP Implementers | 486 | 3.16 | 1.00 |
| Opt for artificial life-sustaining treatment to demonstrate filial piety | ACP Implementers | 486 | 3.01 | .85 |
| Lack of clear guidelines on proper documentation | ACP Implementers | 486 | 2.86 | 1.02 |
| Lack of visible support and buy-in by leaders | ACP Implementers | 486 | 2.81 | 1.02 |
| Increase the risk of higher re-admission rate | ACP Implementers | 486 | 2.42 | .85 |
| Client-related |  |  |  |  |
| Unrealistic expectations of the outcomes of medical treatments | ACP Implementers | 486 | 3.08 | .84 |
| Difficulties in identifying, choosing and engaging nominated spokesperson | ACP Implementers | 486 | 3.05 | .89 |
| Afraid that an ACP will limit the care they will receive | ACP Implementers | 486 | 2.94 | .89 |
| Process: Pre-ACP |  |  |  |  |
| Not emotionally ready to talk about death and dying | HCP (non-ACP) | 98 | 3.34 | .76 |
| Confusion over the different types of ACP | All | 584 | 3.31 | 1.02 |
| Lack of standardised ACP referral criteria | All | 584 | 3.25 | .96 |
| Request to conceal prognosis hinders ACP | HCP (non-ACP) | 98 | 3.21 | .94 |
| Hold unrealistic expectations about recovery | HCP (non-ACP) | 98 | 3.16 | .96 |
| Insufficient coverage on the application of soft skills under training | ACP Implementers | 486 | 3.12 | 1.00 |
| Failure to share about ACP resulted in high rejection | All | 584 | 3.10 | .88 |
| Insufficient contextualisation of knowledge to support application | ACP Implementers | 486 | 2.95 | .91 |
| Expect HCP to avert death | HCP (non-ACP) | 98 | 2.83 | .96 |
| Training is insufficient in equipping knowledge | ACP Implementers | 486 | 2.70 | .93 |
| Opt to pursue aggressive treatments | HCP (non-ACP) | 98 | 2.66 | .86 |
| Low priority among other clinical demands | HCP (non-ACP) | 98 | 2.61 | 1.08 |
| At odds with a physician’s professional goals | HCP (non-ACP) | 98 | 2.61 | .98 |
| Akin to abandoning hope in their treatments | HCP (non-ACP) | 98 | 1.92 | .75 |
| Process: Intra-ACP |  |  |  |  |
| Find ACP facilitation to be complex and time-consuming | ACP Implementers | 486 | 3.37 | .98 |
| Lack of post-training support for facilitators | ACP Implementers | 486 | 3.36 | .96 |
| ACP infrastructure unable to support tracking of adherence | ACP Implementers | 486 | 3.31 | .89 |
| ACP infrastructure does not allow uploading partially completed ACP | ACP Implementers | 486 | 3.30 | .88 |
| ACP infrastructure unable to track changes in care preferences over time | ACP Implementers | 486 | 3.20 | .90 |
| ACP facilitation is emotionally draining for facilitators | ACP Implementers | 486 | 3.19 | .93 |
| Physicians may refuse to endorse ACP decisions | ACP Implementers | 486 | 2.93 | .88 |
| Lack of alignment between clients and nominated spokesperson on decisions | ACP Implementers | 486 | 2.82 | .77 |
| Difficult to build rapport and trust with clients and their families | ACP Implementers | 486 | 2.63 | .82 |
| May not document or upload ACP if my organisation is the sole provider | ACP Implementers | 486 | 2.55 | .98 |
| Process: Post-ACP |  |  |  |  |
| Lack of guidelines on use of completed ACP documentation | All | 584 | 3.18 | .88 |
| Lack of clear workflows and instructions on how to act on ACP preferences | All | 584 | 3.17 | .94 |
| Physicians fail to contextualise documented ACP to current scenario | All | 584 | 3.09 | .82 |
| Find the value of documented ACP is unclear scenarios | All | 584 | 3.08 | .96 |
| ACP clients and/or nominated spokespersons are unable to recall content or documented preferences | All | 584 | 3.05 | .82 |
| Difficult to retrieve ACP documents in emergency | All | 584 | 2.99 | .99 |
| 7.6 Questionable value given the dynamic nature and changing preferences | All | 584 | 2.94 | 1.00 |
| 7.7 Find little value in completing ACP with misalignment in care goals | All | 584 | 2.94 (1.05) | 1.05 |

**ACP Implementers: All full-time and part-time certified ACP facilitators who are doctors, nurses, social workers or others. HCP(Non- ACP): Healthcare professionals who do not have specific ACP implementer roles, but would refer suitable patients for ACP or execute ACP-informed care plans.*
